# Supplementary material for: Clinical academic research in the time of Corona: A simulation study in England and a call for action
Source: PLoS One. 2020 Aug 13;15(8):e0237298. doi: 10.1371/journal.pone.0237298 (PMC7425844; doi:10.1371/journal.pone.0237298)
Supplement: S1 File — (DOCX) [file pone.0237298.s001.docx]

**Web appendix**

*Simulation study for population infection rate*

Our simulation with 640 000 hypothetical individuals (1/100 of the population in the UK) started on the 5 of March, when 23 people had COVID-19 and the rest were healthy(17). We selected 23, because we selected the first date that the confirmed cases were >100 in worldometer (116 cases on the 5/3). So, we assume that on that date, we had 116*20~= 2300 cases, so we used in our simulation 2300/100=23.

The probability$P\left( new case at day n \right)$ is equal to

$$P\left( new case at day n \right)= \left\{ \begin{aligned} 0 if diseased or already infected \\ \frac{new confirmed cases of day n}{uninfected population [day \left( n-1 \right)]} if not diseased or already infected \end{aligned} \right.$$

However, we have that $\frac{new confirmed cases of day n}{uninfected population [day \left( n-1 \right)]}=$

$$\frac{active cases of day \left( n-1 \right) in the simulated dataset}{uninfected population (n-1)}*\frac{new confirmed cases of day n}{active confirmed cases of day \left( n-1 \right)}$$

We inserted values for the ratio $\frac{new confirmed cases of day n}{active confirmed cases of day \left( n-1 \right)}$ from official statistics(17) from day 1 (March 5) to day 36 (April 10). Then we assumed different scenarios that are illustrated in web table 1.

*Effect of the population infection rate on the medical academic capacity*

We made the following assumptions:

1. The number of academics dropped linearly from 3200 to 2400 between day 0 and day 36, because 800 (25% were self-isolated on the 10 April). We further assumed that 800 academics will be quarantined from day 36 to day 90 and will return to work linearly until day 150.
2. The number of clinical academics available on 10 April was 400.
3. We modelled two scenarios for medical academic capacity at 10% (low strain on health system) and 5% (high strain on health system) infection rates respectively, assuming that 1000 clinical researchers would have to work in hospital per 1% of active cases in the population, if the active cases are <2.07%, i.e. infection rate of the 10^th^ of April). In the 10% and 5% cut-off models respectively, we assumed ~50 (i.e. 400/7.93) 2) and ~136 (i.e. 400/2.93) extra researchers can be available per 1% of active cases (≥2.07% i.e. infection rate on 10 April).

**S1 Table 1. Infection growth rate at different times in the pandemic in four different scenarios***

|  | Days 37-50 | Days 51-80 | Days 80-120 | Days 120-250 |
| --- | --- | --- | --- | --- |
| Italy model | Italy’s ratio with 14 days lag | 5% | 5% | 4% |
| Mitigation | 7.5% | 7.4-4.5%  (drop 0.1% per day) | 5% | 5% |
| Relaxed mitigation | From 9.9 to 8.5% (dropping 0.1% per day) | 8.45-7%  (dropping 0.05% per day) | 6.5% | 6.5% |
| Do nothing | 12% | 11.8-6%  (dropping 0.2% per day) | 6% | 6% |

**The values in the table above correspond to the ratio* $\frac{new confirmed cases of day n}{active confirmed cases of day \left( n-1 \right)}$
